# Sales of antimicrobials for veterinary use, Bangladesh, 2023

**DOI:** 10.2471/BLT.25.294366

**Published:** 2026-06-15

**Authors:** SM Sabrina Yesmin, Delfy Góchez, Tanvir Ahmed, Mohammad Nayeem Golder, Pondpan Suwanthada, Niels Frederik Fabricius, Mahbub Hossain, ATM Golam Kibria Khan, Romel Mullick, Md Shariful Islam, Umme Habiba, Aninda Rahman, Md Ashraf Hossain, Md Shameem Haidar

**Affiliations:** a Directorate General of Drug Administration, Ministry of Health and Family Welfare, Aushad Bhaban, 46 Mohakhali, Dhaka-1212, Bangladesh.; b World Organisation for Animal Health, Paris, France.; c School of Public Health, Adelaide University, Adelaide, Australia.; d The Danish Veterinary, Food, Agriculture and Fisheries Agency, Copenhagen, Denmark.; e Directorate General of Health Services, Ministry of Health and Family Welfare, Dhaka, Bangladesh.

## Abstract

**Objective:**

To estimate the volume and patterns of antimicrobial sale for food-producing animals in Bangladesh and to provide evidence to support national policy decisions.

**Methods:**

We used the World Organisation for Animal Health tool (animal antimicrobial use calculation module) for data collection and calculation of quantities of antimicrobials used in animals. We collected data on the overall amount of antimicrobials sold for use in food-producing animals, disaggregated by antimicrobial class, active molecule and route of administration.

**Findings:**

In 2023, 475 750 kg of antimicrobials were sold for use in animals across 301 products and 38 molecules. Oral administration accounted for 95.91% (456 301/475 750) of total antimicrobials sold. Tetracyclines were the most sold antimicrobial class, accounting for 32.22% (153 274/475 750) of antimicrobials sold for use in food-producing animals. The second most-sold class was aminoglycosides at 19.77% (94 044/475 750) of total sales. As regards to active molecules, chlortetracycline, neomycin, ceftriaxone, oxytetracycline, metronidazole, sulfamethoxazole, ciprofloxacin and enrofloxacin accounted for 79.34% (377 438/475 749) of the total national antimicrobial sale. Just over one fifth of the antimicrobials sold belong to classes considered to be highest priority critically important antimicrobials for humans (according to the World Health Organization’s list). These classes were third- and fourth-generation cephalosporins, fluoroquinolones, other quinolones and polymyxins.

**Conclusion:**

Our findings highlight antimicrobial overuse and misuse in food-producing animals in Bangladesh and the need for policy interventions to combat antimicrobial resistance. Our report provides baseline data to support Bangladesh’s *National action plan on antimicrobial resistance* and enable evidence-based policy development and strengthening of antimicrobial stewardship in the veterinary sector.

## Résumé

**Objectif:**

Estimer le volume et les tendances des ventes d’antimicrobiens chez les animaux destinés à l’alimentation au Bangladesh et fournir des données factuelles pour étayer les décisions politiques nationales.

**Méthodes:**

Nous avons utilisé l’outil de l’Organisation mondiale de la santé animale (module de calcul de l’utilisation d’antimicrobiens chez les animaux) pour la collecte de données et le calcul des quantités d’antimicrobiens employées chez les animaux. Nous avons recueilli des données sur les ventes totales d’antimicrobiens pour animaux destinés à l’alimentation, ventilées par classe d’antimicrobiens, principe actif et voie d’administration.

**Résultats:**

En 2023 ont été vendus 475 750 kg d’antimicrobiens pour animaux, répartis en 301 produits et 38 principes actifs. L’administration par voie orale représentait 95,91% (456 301 sur 475 750) du total des antimicrobiens vendus. Les tétracyclines constituaient la classe d’antimicrobiens la plus vendue, se montant à 32,22% (153 274 sur 475 750) des ventes d’antimicrobiens pour animaux destinés à l’alimentation. La deuxième classe d’antimicrobiens la plus vendue était celle des aminoglycosides, avec 19,77% (94 044 sur 475 750) des ventes totales. En ce qui concerne les principes actifs, la chlortétracycline, la néomycine, la ceftriaxone, l’oxytétracycline, le métronidazole, le sulfaméthoxazole, la ciprofloxacine et l’enrofloxacine représentaient 79,34% (377 438 sur 475 749) des ventes nationales totales d’antimicrobiens. Un peu plus d’un cinquième des antimicrobiens vendus appartiennent à des classes considérées comme des antimicrobiens d’importance critique et de priorité absolue pour l’être humain (selon la liste de l’Organisation mondiale de la Santé). Ces classes comprenaient les céphalosporines de troisième et quatrième générations, les fluoroquinolones, les autres quinolones et les polymyxines.

**Conclusion:**

Nos résultats mettent en évidence les cas d’utilisations excessive et abusive d’antimicrobiens chez les animaux destinés à l’alimentation au Bangladesh, ainsi que la nécessité de mettre en place des mesures politiques pour lutter contre la résistance aux antimicrobiens. Notre rapport fournit des données de référence pour soutenir le Plan d’action national du Bangladesh sur la résistance aux antimicrobiens et permettre l’élaboration de politiques fondées sur des données scientifiques, ainsi que le renforcement de la gestion responsable des antimicrobiens dans le secteur vétérinaire.

## Resumen

**Objetivo:**

Estimar el volumen y los patrones de venta de antimicrobianos para animales destinados a la producción de alimentos en Bangladesh y aportar evidencia que sirva de base para la adopción de decisiones de política nacional.

**Métodos:**

Se utilizó la herramienta de la Organización Mundial de Sanidad Animal (módulo de cálculo del uso de antimicrobianos en animales) para recopilar los datos y calcular las cantidades de antimicrobianos utilizadas en animales. Se recopilaron datos sobre la cantidad total de antimicrobianos vendidos para su uso en animales destinados a la producción de alimentos, desglosados por clase de antimicrobiano, principio activo y vía de administración.

**Resultados:**

En 2023, se vendieron 475 750 kg de antimicrobianos para uso en animales, correspondientes a 301 productos y 38 principios activos. La administración por vía oral representó el 95,91% (456 301/475 750) del total de antimicrobianos vendidos. Las tetraciclinas fueron la clase de antimicrobianos más vendida y representaron el 32,22% (153 274/475 750) de los antimicrobianos vendidos para su uso en animales destinados a la producción de alimentos. La segunda clase más vendida fueron los aminoglucósidos, con un 19,77% (94 044/475 750) del total de las ventas. En cuanto a los principios activos, la clortetraciclina, la neomicina, la ceftriaxona, la oxitetraciclina, el metronidazol, el sulfametoxazol, la ciprofloxacina y la enrofloxacina representaron el 79,34% (377 438/475 749) del total nacional de ventas de antimicrobianos. Algo más de una quinta parte de los antimicrobianos vendidos pertenece a clases clasificadas como antimicrobianos de importancia crítica de máxima prioridad para la medicina humana, según la lista de la Organización Mundial de la Salud. Estas clases fueron las cefalosporinas de tercera y cuarta generación, las fluoroquinolonas, otras quinolonas y las polimixinas.

**Conclusión:**

Los resultados ponen de manifiesto el uso excesivo e inadecuado de antimicrobianos en animales destinados a la producción de alimentos en Bangladesh y la necesidad de adoptar intervenciones de política para combatir la resistencia a los antimicrobianos. Este informe proporciona datos de referencia para respaldar el Plan de Acción Nacional sobre la Resistencia a los Antimicrobianos de Bangladesh y favorecer la formulación de políticas basadas en la evidencia, así como el fortalecimiento de los programas de optimización del uso de los antimicrobianos en el sector veterinario.

## ملخص

**الغرض:**

تقدير حجم وأنماط مبيعات مضادات الميكروبات للاستخدام في الحيوانات المنتجة للغذاء في بنغلاديش، وتوفير أدلة تدعم قرارات السياسة الوطنية.

**الطريقة:**

قمنا باستخدام أداة المنظمة العالمية لصحة الحيوان (وحدة حساب استخدام مضادات الميكروبات في الحيوانات) لجمع البيانات وحساب كميات مضادات الميكروبات المستخدمة في الحيوانات. وجمعنا بيانات عن إجمالي كمية مضادات الميكروبات المباعة للاستخدام في الحيوانات المنتجة للغذاء، مصنفة حسب فئة مضاد الميكروبات، والجزيء الفعال، وطريقة الإعطاء.

**النتائج:**

في عام 2023، تم بيع 475750 كيلوجرامًا من مضادات الميكروبات للاستخدام في الحيوانات، موزعة على 301 منتجًا و38 جزيئًا. شكّل الإعطاء عن طريق الفم نسبة %95.91 (456301/475750) من إجمالي مضادات الميكروبات المباعة. كانت التتراسيكلينات الفئة الأكثر مبيعًا من مضادات الميكروبات، حيث شكّلت نسبة %32.22 (153274/475750) من إجمالي مضادات الميكروبات المباعة للاستخدام في الحيوانات المنتجة للغذاء. وكانت الأمينوجليكوزيدات الفئة الثانية الأكثر مبيعًا بنسبة %19.77 (94044/475750) من إجمالي المبيعات. أما بالنسبة للجزيئات الفعالة، فقد شكلت الكلورتتراسيكلين، والنيومايسين، والسيفتركسون، والأوكسيتتراسيكلين، والميترونيدازول، والسلفاميثوكسازول، والسيبروفلوكساسين، والإينروفلوكساسين فقد مثلت %79.34 (377438/475749) من إجمالي مبيعات مضادات الميكروبات على المستوى الوطني. وينتمي ما يزيد قليلاً عن خُمس مضادات الميكروبات المباعة إلى فئات تُعتبر ذات أولوية قصوى وأهمية بالغة للبشر (وفقًا لقائمة منظمة الصحة العالمية). شملت هذه الفئات السيفالوسبورينات من الجيلين الثالث والرابع، والفلوروكينولونات، والكينولونات الأخرى، والبوليميكسينات.

**الاستنتاج:**

تركّز نتائجنا على الإفراط في استخدام، وسوء استخدام، المضادات الحيوية في الحيوانات المنتجة للغذاء في بنغلاديش، والحاجة إلى تدخلات سياسية لمكافحة مقاومة المضادات الحيوية. يوفر تقريرنا بيانات أساسية لدعم خطة العمل الوطنية في بنغلاديش الخاصة بمقاومة المضادات الحيوية، وتمكين تطوير السياسات القائمة على الأدلة، وتعزيز الإشراف على المضادات الحيوية في القطاع البيطري.

## 摘要

**目的:**

估测孟加拉国用于食用动物的抗微生物药物的销售量和销售模式，并为国家政策决策提供循证支持。

**方法:**

我们使用世界动物卫生组织的工具（动物抗微生物药物用量计算模块）来收集数据，计算用于动物的抗微生物药物的数量。我们收集了关于用于食用动物抗微生物药物的售出总量的数据，并按抗微生物类别、活性分子和给药路径进行汇总。

**结果:**

2023 年，有 475750 千克的抗微生物药物售出并用于动物，包括 301 种产品和 38 种分子。口服给药占抗微生物药物总售出量的 95.91% (456301/475750)。四环素类药物是售出最多的抗微生物类药物，占用于食用动物抗微生物药物售出量的 32.22% (153274/475750)。销量第二的类别是氨基糖苷类药物，占总销量的 19.77% (94044/475750)。就活性分子而言，金霉素、新霉素、头孢曲松、土霉素、甲硝唑、磺胺甲噁唑、环丙沙星、恩诺沙星占抗微生物药物总销量的 79.34% (377438/475749)。略超五分之一售出的抗微生物药物属于被认为是对人类具有最高优先级的极重要的抗微生物药物类别（根据世卫组织列表）。这些药物类别是第三代和第四代头孢菌素类、氟喹诺酮类、其他喹诺酮类以及多黏菌素类。

**结论:**

我们的研究结果着重指出了孟加拉国食用动物抗微生物药物过度使用与滥用的问题，需要有相关政策干预措施来应对抗微生物药物耐药性。我们的报告为支持孟加拉国国家抗微生物药物耐药行动计划提供了基准数据，并使之得以制定循证政策，加强兽医领域抗微生物药物的管控。

## Резюме

**Цель:**

Оценить объем и структуру продаж противомикробных препаратов для сельскохозяйственных животных в Бангладеш и предоставить данные для обоснования решений в области национальной политики.

**Методы:**

Для сбора данных и расчета количества противомикробных препаратов, используемых у животных, авторы использовали инструмент Всемирной организации по охране здоровья животных (модуль расчета применения противомикробных препаратов у животных). Были собраны данные об общем количестве противомикробных препаратов, проданных для использования у сельскохозяйственных животных, с разбивкой по классам таких препаратов, активным молекулам и способам введения.

**Результаты:**

В 2023 году было продано 475 750 кг противомикробных препаратов для использования у животных (301 препарат и 38 молекул). На пероральное введение пришлось 95,91% (456 301/475 750) от общего объема проданных противомикробных препаратов. Наиболее продаваемым классом противомикробных препаратов были тетрациклины. Они составили 32,22% (153 274/475 750) от объема препаратов, реализованных для использования у сельскохозяйственных животных. Вторым наиболее продаваемым классом стали аминогликозиды. Они составили 19,77% (94 044/475 750) от общего объема продаж. Что касается активных молекул, на долю хлортетрациклина, неомицина, цефтриаксона, окситетрациклина, метронидазола, сульфаметоксазола, ципрофлоксацина и энрофлоксацина пришлось 79,34% (377 438/475 749) от общего национального объема продаж противомикробных препаратов. Чуть более пятой части реализованных противомикробных препаратов относятся к классам, которые считаются жизненно необходимыми и важнейшими противомикробными препаратами для человека (в соответствии с перечнем Всемирной организации здравоохранения). К этим классам относились цефалоспорины третьего и четвертого поколения, фторхинолоны, другие хинолоны и полимиксины.

**Вывод:**

Полученные результаты указывают на избыточное и нерациональное использование противомикробных препаратов у сельскохозяйственных животных в Бангладеш, а также на необходимость внедрения мер нормативно-правового регулирования для борьбы с устойчивостью к противомикробным препаратам. Данный отчет предоставляет базовые данные для поддержки Национального плана действий по устойчивости к противомикробным препаратам в Бангладеш и делает возможным разработку политики на основе фактических данных, а также усиление контроля за использованием противомикробных препаратов в ветеринарном секторе.

## Introduction

Widespread use of antimicrobials in humans and animals can lead to antimicrobial resistance and multidrug resistance in microorganisms. Estimating antimicrobial use, particularly in the veterinary sector, is important to reduce antimicrobial resistance, as this information provides the evidence needed to guide stewardship policies and regulatory interventions. Surveillance of antimicrobial resistance and antimicrobial use is essential to understand and address the problem and its impact on public health,^1^ as antimicrobial resistance and antimicrobial use are highly correlated.^2^ From an economic perspective, a loss in global gross domestic product of 575 billion United States dollars in food-producing animals due to antimicrobial resistance is projected by 2050.^3^

The indiscriminate use of antimicrobials in animals may be a key contributing factor to bacterial antimicrobial resistance.^4^ However, surveillance and data collection for antimicrobial use are insufficient,^5^ highlighting the global gap in reliable, country-level estimates of antimicrobial consumption in animals. Monitoring antimicrobial use in animals is essential to track progress in reducing farming’s unnecessary reliance on antimicrobials and to identify gaps in antimicrobial stewardship to combat antimicrobial resistance.^6^ Global monitoring is also necessary to ensure a One Health approach,^7^ where interactions occur between human, animal and environmental health.^8^ In the ninth round of data collection, the World Organisation for Animal Health (WOAH) report highlighted several challenges to conducting surveillance of antimicrobial use across countries.^9^ These challenges include a lack of information technology tools and human resources; limited coordination between national authorities and the private sector; inadequate regulatory frameworks; insufficient regulatory enforcement; and political instability.^9^ Currently, most existing global estimates rely on modelled or aggregated data, with limited representation from low- and middle-income countries, including Bangladesh. This situation limits the ability of policy-makers to develop policies based on national evidence. 

Antimicrobial use in livestock systems in South-East Asia is high and driven by farm profitability, disease prevention and mortality reduction.^10^ A 2023 literature review showed that the widespread use of medically important antibiotics is high in South-East Asia in livestock, poultry and aquaculture fish.^11^

Bangladesh has about 402.56 million livestock^12^ and 0.15 million commercial poultry farms, and 94.17% (113/120) of farmers use antibiotics in their farms.^13^ As in other low- and middle-income countries, Bangladesh industries producing terrestrial and aquatic food-animals are increasing to meet the growing demand for animal-source nutrition, resulting in increased use of antimicrobial medicines.^14^ This rising demand further reinforces the need for systematic quantification of veterinary antimicrobial use to inform national and global strategies to reduce antimicrobial resistance. A study on meat-producing animals in Bangladesh found worrying prevalence rates of *Salmonella* spp. contamination in meat samples which demonstrated multidrug resistance. This resistance could be transmitted to humans through food or via environmental spread.^15^

Bangladesh is a medicine-manufacturing country and most of its pharmaceutical demand is met through local production. While this situation ensures accessibility, it also contributes to the widespread availability of antimicrobials, which may facilitate irrational use and environmental contamination, further accelerating the development of antimicrobial resistance.^16^ Additionally, due to inadequate microbial diagnostics facilities in Bangladesh, veterinarians empirically treat animals with antibiotics based on a presumptive diagnosis.^17^ Despite these risks, Bangladesh currently lacks comprehensive, nationally representative data on veterinary antimicrobial use. 

The National Strategy and Action Plan for Antimicrobial Resistance Containment in Bangladesh (2023–2028) emphasizes the establishment of national surveillance of antimicrobial use for veterinary medicine.^18^ The Directorate General of Drug Administration, as the national regulatory authority of drugs, took the initiative to conduct surveillance of veterinary antimicrobial use in collaboration with the Department of Livestock Services. The objective of this surveillance study was to use antimicrobial sales data as a proxy to estimate the volume and patterns of antimicrobial use in food-producing animals in Bangladesh, to provide evidence to support national policy decisions to contain antimicrobial overuse and to contribute to the global reporting efforts.

## Methods

To undertake the surveillance of national sales of antimicrobials for veterinary use, we used the WOAH tool (Animal antimicrobial use calculation module).^19^ This tool allows the collection and calculation of quantities of antimicrobials used in animals, and provides standardized processes for monitoring patterns of antimicrobial use in animals. For data collection and calculation, we applied WOAH reporting option 3,^9^^,^^19^ which represents the overall amount of antimicrobials sold for and/or used in animals by antimicrobial class, with the possibility to separate by type of use, species group and route of administration.

We collected data using pharmaceutical (local manufacturers and importers) sales distribution channels through wholesalers for the year 2023. We collected the sales data from the distribution channels of 29 local manufacturers and importers. These data represent an estimated 90% of the total registered veterinary antimicrobial products in the country, and constitute the most comprehensive nationally available data set on veterinary antimicrobial supply.

For products containing multiple antimicrobial agents (fixed-dose combinations), we disaggregated each active ingredient and quantified them separately according to its declared strength on the product label. Thereafter we classified the product into its respective antimicrobial class following WOAH guidelines.^19^

Antibiotic use for growth promotion of animals is not allowed in Bangladesh; therefore, only therapeutic uses are reported to WOAH. We reported quantities in kilograms of chemical compound as stated on the product label. The estimation process was as follows: (i) we obtained raw sales data (number of units sold per product); (ii) we converted these data into quantities (kg) of active antimicrobial ingredient using product strength; and (iii) we aggregated the total quantities across products and antimicrobial classes to produce national estimates. We converted sales volumes (e.g. number of units sold) into total active ingredient quantities by multiplying the unit strength by the number of units sold. When data were provided in international units, we used standardized conversion factors to express quantities in kilograms of active ingredient.^20^ We aggregated total antimicrobial volumes by antimicrobial class and animal sector.

We standardized information on antimicrobial agents used in animals relative to animal biomass using the following equation: antimicrobial use (mg/kg) = total amount of antimicrobial agents (mg)/total animal biomass (kg).

### Biomass calculation

We calculated animal biomass using the WOAH information system and the Food and Agriculture Organization of the United Nations (FAO) statistics data sets, which represent the total weight of animals exposed to antimicrobial use.^21^ Specifically, we estimated animal biomass by multiplying the number of animals in each category by standard average live weights as defined in WOAH guidelines. Table 1 gives animal population data and biomass data for 2023 for Bangladesh. 

**Table 1:** Animal biomass by animal group and category, Bangladesh, 2023

Animal group and category	Live animals, no.	Slaughtered animals, no.	Meat weight, kg	Mean live weight, kg	Biomass, kg^a^
**Terrestrial **					
Broiler chickens	319 689 000	323 992 000	226 753.2	1.0	323 992 000
Buffalo	1 516 000	88 025	7 042.0	148.1	224 519 600
Cattle	24 856 000	2 792 498	203 220.5	134.8	2 812 819 298
Ducks	66 016 000	64 156 000	64 155.6	1.4	89 818 400
Goats	26 945 000	13 472 500	240 782.4	38.0	1 201 747 000
Sheep	3 827 000	1 033 300	6 199.8	12.8	242 846 240
**Aquatic**					
Crustaceans (aquaculture)	NA	NA	165 937.0	NA	165 937 000
Fish (aquaculture)	NA	NA	2 407 847.0	NA	2 407 847 000
**Total**	NA	NA	NA	NA	**7 469 526 538**

NA: not applicable.

^a^ We estimated biomass using World Organisation for Animal Health methodology and species-specific approaches.^21^ For long-lived livestock (cattle, buffalo, goats and sheep), we derived biomass from census data combined with weighted mean live weights reflecting production structure and slaughter data. For short-cycle species (broiler chickens and ducks), we calculated biomass from annual slaughter numbers multiplied by mean live weight at slaughter. For species with breeding components, biomass includes both slaughtered animals and retained breeding stock using standard assumptions.

Note: The data source was the Food and Agriculture Organization of the United Nations.^22^

As the collected data set represented about 90% of national sales, we extrapolated to 100% coverage following the WOAH tool guide by applying a proportional scaling factor.^19^ This approach assumes that the missing 10% of data follow a similar distribution pattern to the reported data.

## Results

### Biomass and antimicrobial quantity

The total estimated animal biomass was 7 469 526 538 kg based on 2023 FAO data sets (Table 1). The total sales of antimicrobial quantity of active ingredient adjusted to 100% market coverage was 475 750 kg. Dividing this quantity by animal biomass gives the amount of antimicrobials used per animal biomass (mg/kg) which enables comparison across countries and over time. Thus, the overall amount of antimicrobials used per animal biomass was 63.69 mg/kg.

In 2023, antimicrobials sold for veterinary use in Bangladesh included 301 registered products containing 38 antimicrobial molecules. Table 2 presents the amounts of veterinary antimicrobials sold by route of administration and class.

**Table 2:** Amount of antimicrobial medicines sold for all animal species, by antimicrobial class and route of administration, Bangladesh, 2023

Antimicrobial class	Amount of medicine sold, kg			
	All routes	Oral route	Parenteral route	Other route
Aminoglycosides^a^	94 044	91 013	2248	97
Amphenicols	6 549	6 549	0	0
Third- and fourth-generation cephalosporins	61 456	53 219	8237	0
Fluoroquinolones	36 851	32 302	4549	0
Lincosamides	4 431	4 299	133	0
Macrolides	25 342	25 342	0	0
Other quinolones	429	0	0	0
Penicillins	15 651	13 527	2124	0
Pleuromutilins	1 453	1 453	0	0
Polymyxins	568	568	0	0
Sulfonamides (including trimethoprim)	50 052	50 052	0	0
Tetracyclines	153 275	152 387	888	0
Others	25 649	25 590	0	59
**Total**	**475 749**	**456 301**	**18 179**	**156**

^a^ The apparent discrepancies in the totals for aminoglycosides and other quinolones are because four veterinary products did not state the route of administration. Therefore, their totals were added to all routes, but they could not be assigned to any specific route of administration.

Note: The data collected represented an estimated 90% of the total registered veterinary antimicrobial products. The amounts were adjusted from 90% to 100% market coverage.

### Route of administration

Overall, oral administration accounted for 95.91% (456 300/475 750) of total antimicrobial use, followed by parenteral administration at 3.82% (18 179/475 750). Other routes accounted for a very small proportion (< 0.50%).

For the oral route of administration, 83.05% (378 973/456 301) of the total oral consumption of antimicrobials was concentrated in five main antimicrobial classes, including tetracyclines (33.40%; 152 387/456 301), aminoglycosides (19.95%; 91 013/456 301), third- and fourth-generation cephalosporins (11.66%; 53 219/456 301), sulfonamides (10.97%; 50 052/456 301) and fluoroquinolones (7.08%; 32 302/456 301).

For the parenteral route, 82.70% (15 034/18 179) of use was dominated by third- and fourth-generation cephalosporins (45.31%; 8237/18 179), fluoroquinolones (25.03%; 4549/18 179) and aminoglycosides (12.36%; 2248/18 179), indicating reliance on critically important antimicrobials for injectable use.

### Antimicrobial class

Tetracyclines were the most sold antimicrobial class in Bangladesh, with 153 274 kg, that is 32.22% (153 274/475 750) of total sales. Among tetracyclines, chlortetracycline was the predominant active ingredient, accounting for 78.64% (120 533/153 274) of total tetracycline sold, followed by oxytetracycline at 17.59% (26 965/153 274) and doxycycline at 3.77% (5777/153 274). The second most-sold class was aminoglycosides with 94 044 kg, that is 19.77% (94 044/475 750) of total sales. The main aminoglycosides sold were neomycin at 94.49% (88 864/94 044), followed by gentamicin at 5.44% (5113/94 044) and spectinomycin at 0.07% (67/94 044). Third- and fourth-generation cephalosporins were the third most-sold class with 61 456 kg, that is 12.92% (61 456/475 750) of total sales, with ceftriaxone comprising 99.97% (61 438/61 456) of this class.

### Active substances

At the molecule level, the most commonly sold antimicrobials were chlortetracycline, neomycin, ceftriaxone, oxytetracycline, metronidazole, sulfamethoxazole, ciprofloxacin and enrofloxacin, which collectively accounted for 79.34% (377 438/475 749) of total national antimicrobial sales (Table 3). The polymyxin colistin sulfate accounted for 0.12% (568.27/475 749.02) of the total antimicrobials sold in 2023. 

**Table 3:** Amount of antimicrobial medicines sold for veterinary use, by class and active ingredient, Bangladesh, 2023

Antimicrobial class and active ingredients approved for animals	Amount of active ingredient sold, kg (% of total sold)	% within class
**Aminoglycosides**		
Gentamicin	5 112.52 (1.07)	5.44
Neomycin	88 863.51 (18.68)	94.49
Spectinomycin	67.12 (0.01)	0.07
Streptomycin	0.31 (0.00)	0.00
Subtotal	94 043.46 (19.77)	NA
**Amphenicols**		
Florfenicol	6 548.33 (1.38)	100.00
Subtotal	6 548.33 (1.38)	NA
**Cephalosporins, third- and fourth-generation**		
Ceftiofur	17.28 (0.00)	0.03
Ceftriaxone	61 437.86 (12.91)	99.97
Subtotal	61 455.14 (12.92)	NA
**Fluoroquinolones**		
Ciprofloxacin	18 282.45 (3.84)	49.61
Danofloxacin	277.37 (0.06)	0.75
Enrofloxacin	13 374.53 (2.81)	36.29
Levofloxacin	200.27 (0.04)	0.54
Marbofloxacin	4 325.95 (0.91)	11.74
Norfloxacin	390.56 (0.08	1.06
Subtotal	36 851.12 (7.75)	NA
**Lincosamides**		
Lincomycin	4 431.38 (0.93)	100.00
Subtotal	4 431.38 (0.93)	NA
**Macrolides**		
Erythromycin	8 997.77 (1.89)	35.51
Tilmicosin	7 387.58 (1.55)	29.15
Tylosin	7 867.63 (1.65)	31.05
Tylvalosin	1 089.24 (0.23)	4.30
Subtotal	25 342.22 (5.33)	NA
**Other quinolones**		
Flumequine	428.84 (0.09)	100.00
Subtotal	428.84 (0.09)	NA
**Others**		
Metronidazole	25 649.06 (5.39)	100.00
Subtotal	25 649.06 (5.39)	NA
**Penicillins**		
Amoxicillin	13 293.95 (2.79)	84.94
Amoxicillin + clavulanic acid	230.19 (0.05)	1.47
Ampicillin	1 326.86 (0.28)	8.48
Clavulanic acid	799.13 (0.17)	5.11
Penicillin G	0.04 (0.00)	0.00
Penicillin G procaine	0.11 (0.00)	0.00
Subtotal	15 650.29 (3.29)	NA
**Pleuromutilins**		
Tiamulin	1 453.55 (0.31)	100.00
Subtotal	1 453.55 (0.31)	NA
**Polymyxins**		
Colistin sulfate	568.27 (0.12)	100.00
Subtotal	568.27 (0.12)	NA
**Sulfonamides (including trimethoprim)**		
Sulfachloropyridazine	6 813.77 (1.43)	13.61
Sulfadiazine	9 562.09 (2.01)	19.10
Sulfadimidine	767.41 (0.16)	1.53
Sulfamethoxazole	22 333.52 (4.69)	44.62
Sulfaquinoxaline	2 458.72 (0.52)	4.91
Sulfathiazole	321.71 (0.07)	0.64
Trimethoprim	7 795.43 (1.64)	15.57
Subtotal	50 052.65 (10.52)	NA
**Tetracyclines**		
Chlortetracycline	120 532.67 (25.34)	78.64
Doxycycline	5 777.34 (1.21)	3.77
Oxytetracycline	26 964.69 (5.67)	17.59
Subtotal	153 274.70 (32.22)	NA
**Total**	**475 749.02 (100.00)**	**NA**

NA: not applicable.

Note: Inconsistencies arise in some values due to rounding.

## Discussion

Surveillance of veterinary antimicrobial medicine sales data is an effective tool to monitor national trends in antimicrobial use, which is important for therapeutic decisions and evaluation of the effect of misuse and overuse of antimicrobials on resistant strains.^13^ No surveillance system of national antimicrobial use for veterinary medicine exists in Bangladesh. Our surveillance report details sales of antimicrobial for veterinary use in Bangladesh for the year 2023.

The Animal Antimicrobial Use Global Database (ANIMUSE) is a digital platform developed by WOAH in 2015 to systematically collect, manage and analyse data on antimicrobial use in animals on an annual basis.^19^ This tool supports countries in the standardized reporting of antimicrobial quantities through a harmonized framework, improves data quality and transparency, and enables better monitoring and assessment of antimicrobial use. As of April 2026, 49 of 133 countries have made their data publicly available in this database, while the remainder have kept their data confidential.^19^ Among the 49 countries, the average antimicrobial use was 44.13 mg/kg. Bangladesh reported 57.32 mg/kg based on a 90% sales coverage, which is higher than the reported average of these countries. However, regional comparisons indicate variability: in 2021, countries in South-East Asia and the Western Pacific reported a higher average level (167.6 mg/kg), reflecting substantially greater use in some regional production systems. Thus, while Bangladesh’s antimicrobial use is lower than some regional estimates, it still falls within the range of countries with moderate antimicrobial consumption globally. With the updated animal biomass for 2023, Bangladesh is ranked 35th among 133 countries as of April 2026.^19^ These findings contribute to global antimicrobial use reporting and fill a data gap for South-East Asia, a region previously underrepresented in global surveillance systems such as the Animal Antimicrobial Use Global Database. 

In 2022, WOAH reported that tetracyclines (28.29%; 19 983 101.01/70 647 931.6 kg) were the most-sold antimicrobials globally, followed by penicillin (17.75%; 12 539 558.43/70 647 931.6 kg) and macrolides (10.62%; 7 505 502.19/70 647 931.6 kg).^9^ Our results further show that the high sales of tetracyclines are largely driven by compounds in the class, particularly chlortetracycline and oxytetracycline. In addition, our results show high sales of neomycin, ceftriaxone, sulfamethoxazole, ciprofloxacin, enrofloxacin and other antimicrobials, collectively accounting for almost 80% of total national antimicrobial sales for veterinary medicine. This finding reflects the widespread use and potential overuse of medically important antimicrobials in the veterinary sector of Bangladesh. Furthermore, despite regulatory restrictions, the sale of colistin sulfate (568.27 kg) was identified in our study, although it was banned for veterinary use by the Directorate-General of Drug Administration following the Drug Control Committee decision in March 2022.^23^ This finding suggests possible continued availability or use through existing supply chains.

To ensure the rational use of antibiotic substances, the World Health Organization (WHO) publishes *Critically important antimicrobials for human medicine*,^24^ focusing on their use in humans, and WOAH publishes *World Organisation for Animal Health list of antimicrobial agents of veterinary importance*,^25^ focusing on their use in animals.^26^ The United Nations General Assembly highlighted the impact of the use of medically important antimicrobials as growth promoters on antimicrobial resistance in 2024.^27^ Additionally, the third global high-level ministerial conference on antimicrobial resistance in November 2022 recommended preserving critically important antimicrobials for human medicine and ending the use of medically important antimicrobials for growth promotion in animals.^28^ Nonetheless, our surveillance study highlights that 20.87% (99 304/475 750) of the total antimicrobial sales belong to classes considered to be among the highest priority critically important antimicrobials in humans (according to the WHO list). These classes in the WOAH online database are third-and fourth-generation cephalosporins, fluoroquinolones, other quinolones and polymyxins.^19^ In this context, ceftriaxone is considered a WHO highest priority critically important antimicrobial, whereas ceftiofur, a closely related third-generation cephalosporin, is used exclusively in veterinary medicine, highlighting the cross-sectoral risk of selection for resistance between human and animal health systems. Ciprofloxacin is likewise classified as a WHO highest priority critically important antimicrobial within the fluoroquinolone class and is restricted to human medicine use. A study assessing trends between 2019 and 2023, demonstrated that the use of amoxicillin, ciprofloxacin, doxycycline, metronidazole, oxytetracycline and sulfamethoxazole is high both in humans and animals in Bangladesh.^29^ The Directorate General of Drug Administration banned 34 antibiotics for veterinary use in line with the recommendations of the 250th and 253rd meetings of the Drug Control Committee. All combinations of amoxicillin, ciprofloxacin and doxycycline were banned, but not single-dosage forms.^23^ This continued use highlights the need for evidence-based policy refinement and stronger regulatory enforcement mechanisms.

Globally, third- and fourth-generation cephalosporins accounted for only 0.87% (615 243.4/70 647 931.6 kg) of total antimicrobial use and ranked 16th in the 2022 WOAH antimicrobial use surveillance report.^9^ In contrast, our study demonstrates that these cephalosporins were the third most commonly sold antimicrobial class in veterinary medicine overall in Bangladesh for all dosage forms. Notably, for parenteral dosage forms, these antimicrobials accounted for almost half of total sales, making them the most frequently sold class in veterinary medicine in Bangladesh.

In an antimicrobial susceptibility test where *Escherichia coli* was isolated from cattle, sheep, chicken and humans, 37.5% of isolates were multidrug-resistant, and in the antimicrobial susceptibility test, *E. coli* showed the highest resistance to sulfamethoxazole–trimethoprim (71.05%; 108/152), tetracycline (62.50%; 95/152) and ampicillin (61.84%; 94/152).^30^ A scoping review identified that easy antibiotic access and limited veterinary care are responsible for the inappropriate use of antibiotics in the veterinary sectors of South-East Asia.^31^ These findings, together with ours, highlight a need for policy-level intervention, field monitoring, regulatory enforcement and an emphasis on establishing adequate microbial diagnostics facilities in Bangladesh.

Our study has several limitations that may influence the accuracy and interpretation of the findings. There is a risk of misclassification of multi-ingredient antimicrobial products, which could lead to overestimation or underestimation of specific antimicrobial classes. The use of antimicrobials as growth promoters, particularly through medicated feed, is not fully captured in sales data, resulting in potential underreporting of antimicrobial use. Notably, we used supply data to estimate antimicrobial use which does not account for actual consumption, off-label use, stockpiling or wastage at the farm level, which may affect the accuracy of our antimicrobial use estimates. Additionally, illegal, unofficial or unregulated market channels were not captured, which again may result in an underestimation of total antimicrobial use. In addition, sales data may not correspond to actual consumption due to possible waste, spoilage or losses within the distribution chain. We were unable to provide species-specific estimates of antimicrobial use because most products were indicated for multiple animal species. Additionally, the data source was based on aggregate sales information, which does not allow for disaggregation or accurate attribution of use at the species level. Our study is further constrained by gaps in the surveillance system, including incomplete reporting and limited integration across sectors. Uncertainties in biomass estimation may also affect the accuracy of our calculations of the amount of antimicrobial used per animal biomass and limit comparability with other countries. For example, the use of standardized average live weights from WOAH guidelines may not capture local variations in breeds, production systems and farming practices in Bangladesh. Furthermore, species allocation based on labelled indications and proportional distribution for multispecies products may not represent actual usage patterns. These factors, combined with reliance on secondary data sources (from WOAH and FAO), may introduce a margin of error in the biomass estimates which should be considered when interpreting antimicrobial use normalized by biomass.^9^ Taken together, these limitations suggest that the reported antimicrobial use values should be interpreted with caution, as they may either underestimate or overestimate the true level of antimicrobial exposure in animal populations in Bangladesh.

To conclude, our findings provide important evidence on current antimicrobial use in the animal health sector in Bangladesh. The findings highlight the substantial use of tetracyclines, aminoglycosides, third- and fourth-generation cephalosporins, sulfonamides and fluoroquinolones, indicating widespread reliance on medically important antimicrobials for humans.

Importantly, this report provides a baseline data set to support Bangladesh’s National Action Plan on Antimicrobial Resistance and enable evidence-based policy development, prioritization of regulatory interventions and strengthening of antimicrobial stewardship in the veterinary sector. The Directorate General of Drug Administration, Department of Livestock Services and other veterinary authorities can use the results to inform their efforts to refine control measures, update essential drug policies, restrict the use of inappropriate antimicrobial classes and improve enforcement mechanisms, particularly for critically important antimicrobials. In addition, the data establish a baseline for monitoring progress over time and support Bangladesh’s integration into global reporting frameworks such as WOAH’s Animal Antimicrobial Use Database. Finally, the findings underscore the need for coordinated One Health actions involving regulatory authorities, veterinary practitioners, feed industries and farmers to promote prudent and responsible antimicrobial use in veterinary practice and reduce the risk of the emergence of antimicrobial resistance and transmission between animals, humans and the environment in Bangladesh. These actions should be aligned with WHO antimicrobial classification and stewardship principles to preserve the effectiveness of critically important antimicrobials for humans.
